# Analysis of ethanol fermentation mechanism of ethanol producing white-rot fungus *Phlebia* sp. MG-60 by RNA-seq

**DOI:** 10.1186/s12864-016-2977-7

**Published:** 2016-08-11

**Authors:** Jianqiao Wang, Tomohiro Suzuki, Hideo Dohra, Shoko Takigami, Hiroko Kako, Ayumi Soga, Ichiro Kamei, Toshio Mori, Hirokazu Kawagishi, Hirofumi Hirai

**Affiliations:** 1 Faculty of Agriculture, Shizuoka University, 836 Ohya, Suruga-ku, Shizuoka 422-8529 Japan; 2 Center for Bioscience Research and Education, Utsunomiya University, 350 Mine-machi, Utsunomiya, 321-8505 Japan; 3 Institute for Genetic Research and Biotechnology, Shizuoka University, 836 Ohya, Suruga-ku, Shizuoka 422-8529 Japan; 4 Research Institute of Green Science and Technology, Shizuoka University, 836 Ohya, Suruga-ku, Shizuoka 422-8529 Japan; 5 Faculty of Agriculture, University of Miyazaki, 1-1 Gakuen-kibanadai-nishi, Miyazaki, 889-2192 Japan; 6 Graduate School of Science and Technology, Shizuoka University, 836 Ohya, Suruga-ku, Shizuoka 422-8529 Japan

**Keywords:** RNA-seq, Ethanol fermentation mechanism, *Phlebia* sp. MG-60, White-rot fungi

## Abstract

**Background:**

The white-rot fungus *Phlebia* sp. MG-60 shows valuable properties such as high ethanol yield from several lignocellulosic materials, although white-rot fungi commonly degrade woody components to CO_2_ and H_2_O. In order to identify genes involved in ethanol production by *Phlebia* sp. MG-60, we compared genes differentially expressed by the ethanol producing fungus *Phlebia* sp. MG-60 and the model white-rot fungus *Phanerochaete chrysosporium* under ethanol fermenting and non-fermenting conditions using next-generation sequencing technologies.

**Results:**

mRNAs from mycelia of *Phlebia* sp. MG-60 and *P. chrysosporium* under fermenting and non-fermenting conditions were sequenced using the MiSeq system. To detect differentially expressed genes, expression levels were measured in fragments per kilobase of exon per million mapped reads (FPKM). Differentially expressed genes were annotated using BLAST searches, Gene Ontology classifications, and KEGG pathway analysis. Functional analyses of differentially expressed genes revealed that genes involved in glucose uptake, glycolysis, and ethanol synthesis were widely upregulated in *Phlebia* sp. MG-60 under fermenting conditions.

**Conclusions:**

In this study, we provided novel transcriptomic information on *Phlebia* sp. MG-60, and these RNA-seq data were useful in targeting genes involved in ethanol production for future genetic engineering.

**Electronic supplementary material:**

The online version of this article (doi:10.1186/s12864-016-2977-7) contains supplementary material, which is available to authorized users.

## Background

Most bioethanol production in the world is currently from food crops, which leads to competition with food and feed uses. Advanced second-generation bioethanol is a renewable transportation fuel made from lignocellulosic biomass that does not compete with food or feed [1]. Lignocellulosic biomass is mainly composed of cellulose, hemicellulose, and lignin and is the most abundant material for bioethanol production. Wood-rot basidiomycetes play key roles in the carbon cycle in forest ecosystems through multi-enzyme systems that degrade lignocelluloses [2]. Wood-rot basidiomycetes are considered the most efficient degraders of lignocellulose in nature. Biological delignification by white-rot basidiomycetes, which is a useful pretreatment for enzymatic saccharification of lignocellulosic biomass, is summarized by Moreno et al. [3]. Basidiomycetes are expected to be used for pretreatment in bioethanol production from lignocellulosic materials. Therefore, some researchers have reported use of white-rot fungi in pretreatment for enzymatic saccharification of lignocellulosic biomass [4–6]. Furthermore, several reports have been published about genome projects on Basidiomycota, Agaricomycetes and Polyporales lignocellulose-degrading fungi, and comparative genomic studies recently [7–10].

Several white-rot fungi have been reported to ferment oligosaccharide materials to ethanol. The white-rot fungi *Peniophora cinerea* and *Trametes suaveolens* efficiently convert hexoses to ethanol [11], and *Trametes hirsuta* shows efficient fermentation of starch, wheat bran and rice straw to ethanol without acid or enzymatic hydrolysis [12]. Recently, Okamoto et al. documented that the white-rot fungus *Trametes versicolor* KT9427 can produce ethanol from starch, cellulose, xylan, wheat bran and rice straw [13].

The white-rot fungus *Phlebia* sp. MG-60 was selected as a hypersaline-tolerant lignin-degrading fungus from 28 mushrooms and samples of driftwood based on decolorization and delignification abilities after collection from mangrove stands in Okinawa, Japan [14]. *Phlebia* sp. MG-60 was capable of converting lignocellulose to ethanol directly with high yield [6, 15, 16]. When this fungus was cultured with 20 g L^−1^ of unbleached hardwood kraft pulp for 168 h, 71.8 % of the theoretical maximum yield of ethanol was observed, and when it was cultured with waste newspaper, 51.5 % of the theoretical maximum yield was observed [16]. The delignification, anaerobic saccharification, and fermentation of oak wood using only *Phlebia* sp. MG-60, without addition of chemicals or enzymes, has been also reported [16]. Additionally, alkaline pretreated sugarcane bagasse was fermented well directly, without addition of cellulases, by *Phlebia* sp. MG-60 [6]. Thus, *Phlebia* sp. MG-60 possesses not only wood degrading ability but also ethanol fermentability. However, the detailed mechanism of fermentation by this fungus remains unknown.

The goal of this study was to characterize the specific genes for ethanol production, and to predict the mechanism behind the high yield of ethanol by *Phlebia* sp. MG-60. In the present study, we analyzed differential gene expression of the ethanol producing white-rot fungus *Phlebia* sp. MG-60 and the model white-rot fungus *P. chrysosporium*, used as the control, under fermenting and non-fermenting conditions by next-generation sequencing. This is the first report of transcriptomic data of the ethanol producing white-rot fungus *Phlebia* sp. MG-60.

## Methods

### Strains

*Phlebia* sp. MG-60 (MKFC40001) and *P. chrysosporium* ME-446 (ATCC 34541) were used in this study. Both strains were maintained on potato dextrose agar (PDA) slants at 4 °C.

### Production of ethanol from glucose

In order to equalize the growth of mycelia, *Phlebia* sp. MG-60 and *P. chrysosporium* were incubated on PDA plates at 30 °C for 5 and 3 days, respectively. 10-mm diameter disks were then punched out from the growing edge of the mycelia using a sterile cork borer. Two mycelial disks for each strain were placed into a 100-mL Erlenmeyer flask containing 10 mL basal liquid medium (20 g L^−1^ glucose, 10 g L^−1^ yeast extract, 10 g L^−1^ KH_2_PO_4_, 2 g L^−1^ (NH_4_)_2_SO_4_, and 0.5 g L^−1^ MgSO_4_-7H_2_O, pH 4.5). After sealing the flask with a silicone plug stopper (to ensure semi-aerobic conditions), the culture was statically incubated at 30 °C for 10 days. Cultures were filtered through a 0.2-μm membrane filter, and the filtrate was then separated by high-performance liquid chromatography (HPLC) using a Shodex SH1821 column (8.0 mm × 300 mm, Showa Denko K.K., Tokyo, Japan) at 75 °C with 0.5 mM H_2_SO_4_ as the mobile phase at a flow rate of 0.6 mL min^−1^, and ethanol and glucose concentrations in the cultures were measured using an online refractive index detector. The pH of the culture was also measured by a glass electrode (D-51S, Horiba Ltd., Kyoto, Japan).

### Mycelial dry weight

For monitoring the growth of *Phlebia* sp. MG-60 and *P. chrysosporium*, mycelial dry weights obtained from liquid culture which described as above were measured. Cultures were filtered through 0.2-μm membrane filter, mycelium and filter were then dried. The mycelial dry weight calculates by (weight of filter + dried residue) – (weight of filter paper).

### cDNA library preparation for DNA sequencing

To construct RNA-seq libraries, total RNA was isolated from the mycelia of *Phlebia* sp. MG-60 after 2 and 9 days of incubation and from mycelia of *P. chrysosporium* after 3 and 9 days of incubation. Total RNA was first purified from cultured mycelia with three biological replicates obtained from separate cultures using a Qiagen RNeasy Mini Kit (Hilden, Germany). The quality and quantity of each RNA sample were assessed as described previously [17]. Agarose gel electrophoresis and the OD260/OD280 ratio were used for assessing quality of total RNA.

Each extracted total RNA sample was treated with DNase I and repurified using an RNeasy Mini Kit (Qiagen) following the manufacturer’s protocol. Purified RNA (1 μg) was used for first-strand cDNA synthesis using an oligo-dT primer and PrimeScript reverse transcriptase (Takara). An equal quantity of 50 ng total RNA was used for PCR. Primer sequences and the expected product sizes are shown in Additional file 1: Table S1. RT-PCR was performed using 1 μg of total RNA with the PrimeScript RT-PCR Kit and the RT-PCR amplified fragments were analyzed by agarose gel electrophoresis. The libraries for strand-specific RNA sequencing were constructed using a SureSelect Strand-Specific RNA Library prep kit (Agilent Technologies) according to the manufacturer's protocol. We generated libraries of each biological replicates, and then libraries derived from same species were pooled together. The two individual sequencing runs for *Phlebia* sp. MG-60 and *P. chrysosporium* were performed, respectively. Transcriptome sequencing of paired-end reads (75 bp) was performed by a MiSeq system (Illumina).

### *De novo* assembly and differential expression analysis

The raw reads were processed using cutadapt version 1.8.1 to remove adapter sequences [18], low-quality bases (quality scores <30) and reads shorter than 50 nt. The last 76 bases were trimmed by FASTX-Toolkit [19]. After quality trimming, the high-quality reads were assembled into unigenes by Trinity (version 2.0.6) [20] Oases (version 0.2.08) [21], Trans-ABySS (version 1.5.4) [22] and SOAPdenovo-Trans (version 1.03) [23]. The resulting unigenes was further analyzed using DETONATE (*de novo* transcriptome RNA-seq assembly with or without the truth evaluation) [24]. In *de novo* transcriptome assembly by Trinity program, we selected the Jaccard-clip option to reduce the generation of chimeric transcripts. rRNA were excluded from the unigenes by removing sequences matching entries in the SILVA rRNA database by the Megablast program [25]. The genes derived from mitochondria sequences were manually removed from the unigenes using the result of local BLASTX and BLASTN search against nr and nt database. High-quality short reads were mapped to the rRNA-removed unigenes as a reference using Bowtie [26], and then transcript abundance was estimated using RSEM software [27]. To identify differentially expressed genes (DEGs), *P*-values and fold changes were computed using the edgeR package [28].

### Functional annotation

The unigenes were searched against the Swiss-Prot database by a local BLASTX algorithm (E-value cut-off was set at 10e-5) to predict the biological functions [29]. Open reading frames (ORFs) and their protein sequences were predicted from unigenes using TransDecoder, which is included in the Trinity package. Kyoto Encyclopedia of Genes and Genomes (KEGG) pathways were assigned to unigenes using the BlastKOALA server [19, 30]. Gene ontology (GO) annotation of the transcriptome was performed using InterproScan software version with the “goterms” option [31]. Orthologous protein pairs between *Phlebia* sp. MG-60 and *P. chrysosporium* were identified by the FastOrtho program (http://enews.patricbrc.org/fastortho/) [32] as described previously [33], which is a reimplementation of the OrthoMCL program [34]. The longest peptides translated by TransDecoder were used for orthologous protein analysis as representatives of peptides encoded by the Trinity unigenes. Proteins showing one-to-one correspondence between *Phlebia* sp. MG-60 and *P. chrysosporium* were regarded as orthologous proteins.

### Semi-quantitative RT-PCR and quantitative RT-PCR (qRT-PCR)

To analyse gene expression in *Phlebia* sp. MG-60, samples were prepared as described in the section *Production of ethanol from glucose*. The semi-quantitative RT-PCR method was as described in our previous study [35]. All the selected unigenes used in RT-PCR and qRT-PCR evidenced high similarity with known genes with very low E-values (< E-20), and these transcripts were manually confirmed mapping rate of sequencing reads from each sample and sequence errors using the visualized software IGV tools version 2.0 (http://software.broadinstitute.org/software/igv/home). PCR was performed for 28 cycles, with template denaturation at 95 °C for 30 s, primer annealing at 58 °C for 30 s, and DNA extension at 72 °C for 1 min using Ex Taq DNA Polymerase (TaKaRa Bio).

For the quantitative real-time RT-PCR assays, the Roche LightCycler 480 system was used. Each reaction contained 50 ng of first-strand cDNAs, 2 μL PCR primers, 7 μL water, and 10 μL master mix (Roche). Cycling conditions were set as follows: pre-incubation, 95 °C for 10 min; amplification, 45 cycles of 95 °C for 10 s, 60 °C for 10 s, and 7 °C for 10 s. The reference genes *actin* and *hydroxymethylbilane synthase* (HMBS) were used to test for sample-to-sample variation. Relative quantitation using the comparative Ct method was calculated as ΔΔCt = (ΔCt_target_ –ΔCt_control_) _fermenting conditions_ − (ΔCt_target_ – ΔCt_control_) _non-fermenting conditions_..

## Results

### Production of ethanol from glucose

**Fig. 1:**
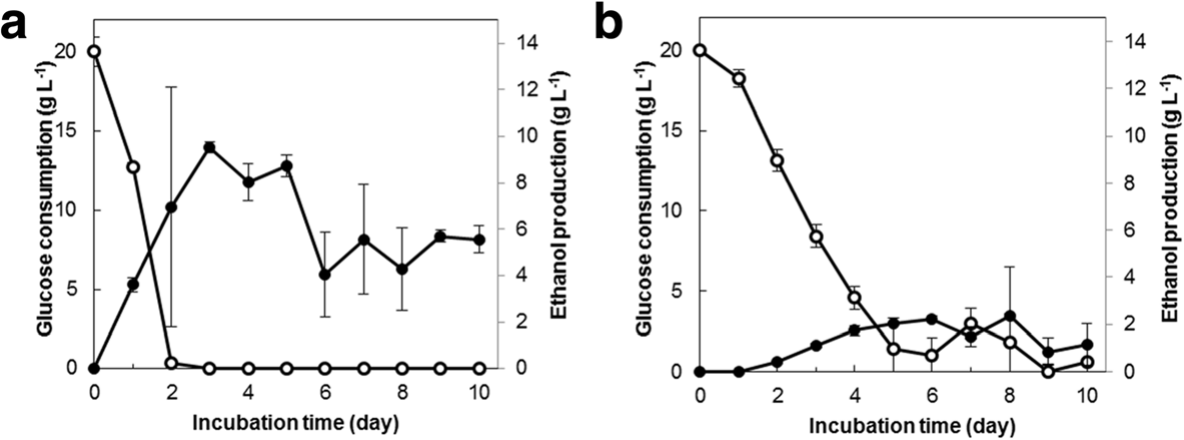
Time courses of ethanol production and glucose consumption of *Phlebia* sp. MG-60 (**a**) and *P. chrysosporium* (**b**). ●: ethanol production; ○: glucose consumption. Values are the means ± SD of triplicate samples

The time courses of ethanol production from glucose and glucose consumption by the ethanol producing white-rot fungus *Phlebia* sp. MG-60 and the control white-rot fungus *P. chrysosporium* are shown in Fig. 1. *Phlebia* sp. MG-60 produced the maximum ethanol concentration of 9.5 g L^−1^ from glucose after 3 days of incubation, and the concentration then gradually diminished. On the other hand, maximum ethanol production by *P. chrysosporium* was 2.4 g L^−1^ after 8 days of incubation (Fig. 1). The glucose concentration of *Phlebia* sp. MG-60 and *P. chrysosporium* were 12.8 g L^−1^ and 18.2 g L^−1^ at 1 day, and then almost decreased to 0 g L^−1^ until 2 days and 5 days after incubation, respectively. We also measured the mycelial growth and pH changes in liquid cultures of both species (Additional file 2: Figure S1). Although the growth rate of *P. chrysosporium* was higher than that of *Phlebia* sp. MG-60, ethanol productivity of *Phlebia* sp. MG-60 was much higher than that of *P. chrysosporium*. The pH in liquid cultures of both species was between 4.5 and 3.8 during the experiment. All the results indicated that there exists a correlation between consumption of glucose and production of ethanol in the mycelia culture of *Phlebia* sp. MG-60 at 2 days after incubation. The consumed glucose was mainly used for its ethanol production in *Phlebia* sp. MG-60. The maximum ethanol productivity was at 2 days in *Phlebia* sp. MG-60 and 3 days of incubation in *P. chrysosporium*, and that ethanol production stopped at 9 days of incubation in both fungi. Therefore, 2-day-incubated mycelia of *Phlebia* sp. MG-60 and 3-day-incubated mycelia of *P. chrysosporium* were used as the fermenting samples, and 9-day-incubated mycelia of *Phlebia* sp. MG-60 and 9-day-incubated mycelia of *P. chrysosporium* were used as the non-fermenting samples for further experiments.

### Sequencing and *de novo* assembly

Total RNA was extracted from *Phlebia* sp. MG-60 and *P. chrysosporium* for transcriptome analysis. Samples included biological triplicates of cDNA libraries from *Phlebia* sp. MG-60 after 2 and 9 days of incubation and *P. chrysosporium* after 3 and 9 days of incubation (termed MG2D, MG9D, PC3D, and PC9D, respectively). Total mRNA was purified, and then cDNA libraries were constructed and sequenced on the MiSeq platform.

Paired-end sequences (2 x 75 bp in length) from mRNAs of *Phlebia* sp. MG-60 and *P. chrysosporium* were generated by MiSeq: 17,822,770 reads (8,911,385 pairs) in MG2D, 25,249,086 reads (12,624,543 pairs) in MG9D, 31,810,504 reads (15,905,252 pairs) in PC3D, and 15,905,252 reads (7,952,626 pairs) in PC9D (Additional file 3: Table S2). After removal of low-quality regions of the reads (quality values <30), each of the high-quality reads from *Phlebia* sp. MG-60 and *P. chrysosporium* were assembled into unigenes using the Trinity, Oases, Trans-ABySS and SOAPdenovo-Trans program, and further analyzed using DETONATE. The resulting RSEM-EVAL score from DETONATE analysis indicated that Trinity produced the most accurate assemblies in all data sets. Next, rRNA and genes derived from mitochondria sequences were excluded from Trinity unigenes by removing those matching entries in the SILVA 111 rRNA [36], nr and nt database. Ultimately, we obtained 34,731 (N50 length = 1,422 bp) unigenes in *Phlebia* sp. MG-60 and 27,956 (N50 length = 1,533 bp) in *P. chrysosporium* (Table 1).

**Table 1:** Assembly summary

	*Phlebia* sp. MG-60	*P. chrysosporium*
Total sequences	34,731	27,956
Total bases	36,064,428	31,633,575
Max sequence length (bp)	9,412	7,982
Average sequence length (bp)	1,038	1,132
Median sequence length (bp)	794	876
N50 length (bp)	1,422	1,533
(A + T)s	45.81 %	41.52 %
(G + C)s	54.19 %	58.48 %

### Functional annotations of differentially expressed genes

All unigenes were searched against the Swiss-Prot database using local BLASTX (Additional file 4: Table S3, Additional file 5: Table S4), and further annotated with GO terms using InterProScan. To identify DEGs, we compared the unigenes that were over 2-fold up- or downregulated with false discovery rate (FDR) < 0.05 between the fermenting and non-fermenting conditions. In *Phlebia* sp. MG-60, 2,944 genes were upregulated, and 3,910 genes were downregulated (Additional file 4: Table S3). There were 1,689 upregulated genes and 1,901 downregulated genes in *P. chrysosporium* (Additional file 5: Table S4). We performed GO enrichment analysis of *Phlebia* sp. MG-60 and *P. chrysosporium*, which revealed that 27 GO terms were upregulated and 5 were downregulated in *Phlebia* sp. MG-60. The most enriched GO terms were “ATP binding” (GO: 0005524) in the molecular functions category, “fatty acid biosynthetic process” (GO: 0006633) in biological processes, and “integral component of membrane” (GO: 0016021) in cellular components under fermenting conditions (Table 2). In contrast, only “catalytic activity” (GO: 0003824) in molecular functions was significantly enriched in *P. chrysosporium* (data not shown).

**Table 2:** Enrichment of GO terms in differentially expressed sequences in *Phlebia* sp. MG-60

GO_name		GO_id	Number of sequences	Log Fold Change	*Z* score	*P*-value	FDR
MF	ATP binding	GO:0005524	799	0.2985	6.1830	6.29E-10	1.4E-07
MF	nucleic acid binding	GO:0003676	233	0.4418	4.6111	4.01E-06	0.0004
MF	phosphogluconate dehydrogenase (decarboxylating) activity	GO:0004616	11	−2.2802	−4.2490	2.15E-05	0.0010
BP	pentose-phosphate shunt	GO:0006098	11	−2.2802	−4.2490	2.15E-05	0.0010
MF	transferase activity	GO:0016740	13	1.9417	4.2346	2.29E-05	0.0010
BP	fatty acid biosynthetic process	GO:0006633	11	2.0965	4.1939	2.74E-05	0.0010
MF	copper ion binding	GO:0005507	27	1.2629	4.0514	5.09E-05	0.0016
BP	tRNA aminoacylation for protein translation	GO:0006418	38	0.9948	3.8449	0.0001	0.0032
MF	aminoacyl-tRNA ligase activity	GO:0004812	41	0.9504	3.8284	0.0001	0.0032
MF	acyl-CoA dehydrogenase activity	GO:0003995	16	−1.7053	−3.7871	0.0002	0.0034
MF	oxidoreductase activity, acting on the CH-CH group of donors	GO:0016627	25	−1.3711	−3.7618	0.0002	0.0034
CC	integral component of membrane	GO:0016021	492	0.2125	3.7428	0.0002	0.0034
BP	intracellular protein transport	GO:0006886	81	0.6338	3.7239	0.0002	0.0034
BP	rRNA processing	GO:0006364	17	1.4230	3.5985	0.0003	0.0051
MF	nucleotide binding	GO:0000166	69	0.6564	3.5460	0.0004	0.0058
MF	nitronate monooxygenase activity	GO:0018580	16	−1.5292	−3.3773	0.0007	0.0102
MF	coenzyme binding	GO:0050662	76	0.5828	3.3485	0.0008	0.0106
BP	biosynthetic process	GO:0009058	73	0.5771	3.2533	0.0011	0.0141
MF	protein binding	GO:0005515	816	0.1157	3.2119	0.0013	0.0154
MF	DNA-directed RNA polymerase activity	GO:0003899	48	0.6911	3.0976	0.0020	0.0217
CC	membrane coat	GO:0030117	15	1.2752	3.0474	0.0023	0.0243
MF	methyltransferase activity	GO:0008168	77	0.5169	3.0342	0.0024	0.0243
MF	RNA binding	GO:0003723	107	0.4216	3.0033	0.0027	0.0256
CC	cytoplasm	GO:0005737	103	0.4287	2.9886	0.0028	0.0256
MF	structural molecule activity	GO:0005198	11	1.4658	2.9773	0.0029	0.0256
MF	DNA binding	GO:0003677	275	0.2301	2.9679	0.0030	0.0256
BP	ribosome biogenesis	GO:0042254	12	1.3692	2.9151	0.0036	0.0282
CC	membrane	GO:0016020	319	0.2020	2.9042	0.0037	0.0282
MF	magnesium ion binding	GO:0000287	27	0.8832	2.9037	0.0037	0.0282
MF	catalytic activity	GO:0003824	327	0.1926	2.8416	0.0045	0.0332
MF	oxidoreductase activity, acting on the aldehyde or oxo group of donors, NAD or NADP as acceptor	GO:0016620	10	1.4184	2.7516	0.0059	0.0425
CC	small-subunit processome	GO:0032040	13	1.2130	2.7064	0.0068	0.0472

*GO* gene ontology, *PAGE* parametric analysis of gene set enrichment, *BP* biological process, *MF* molecular function, *CC* cellular component, *FDR* False discovery rate, Log Fold Change values between fermenting and non-fermenting conditions were used to calculate *Z* scores. Log Fold change values of each GO terms upregulated in fermenting condition is represented by positive numbers and downregulated is represented by negative numbers

To evaluate the high ethanol productivity in *Phlebia* sp. MG-60, we mapped all genes in *Phlebia* sp. MG-60 and *P. chrysosporium* to KEGG metabolic pathways using BlastKOALA, and focused on genes involved in ethanol fermentation. Metabolic pathways and biosynthesis of secondary metabolites were the most frequently represented pathways, including glycolysis and pyruvate oxidation, which are involved in ethanol production pathways. Based on the KEGG pathway assignments, we compared the genes involved in the glycolysis/gluconeogenesis pathway in *Phlebia* sp. MG-60 and *P. chrysosporium* (Additional file 6: Figure S2). Lists of transcripts related to the glycolysis/gluconeogenesis pathway are shown in Additional file 7: Table S5 and Additional file 8: Table S6. In all, 40 genes were mapped to the glycolysis/gluconeogenesis pathway in *Phlebia* sp. MG-60, but only 18 in *P. chrysosporium*.

### Orthologous analysis of *Phlebia* sp. MG-60 and *P. chrysosporium*

**Fig. 2:**
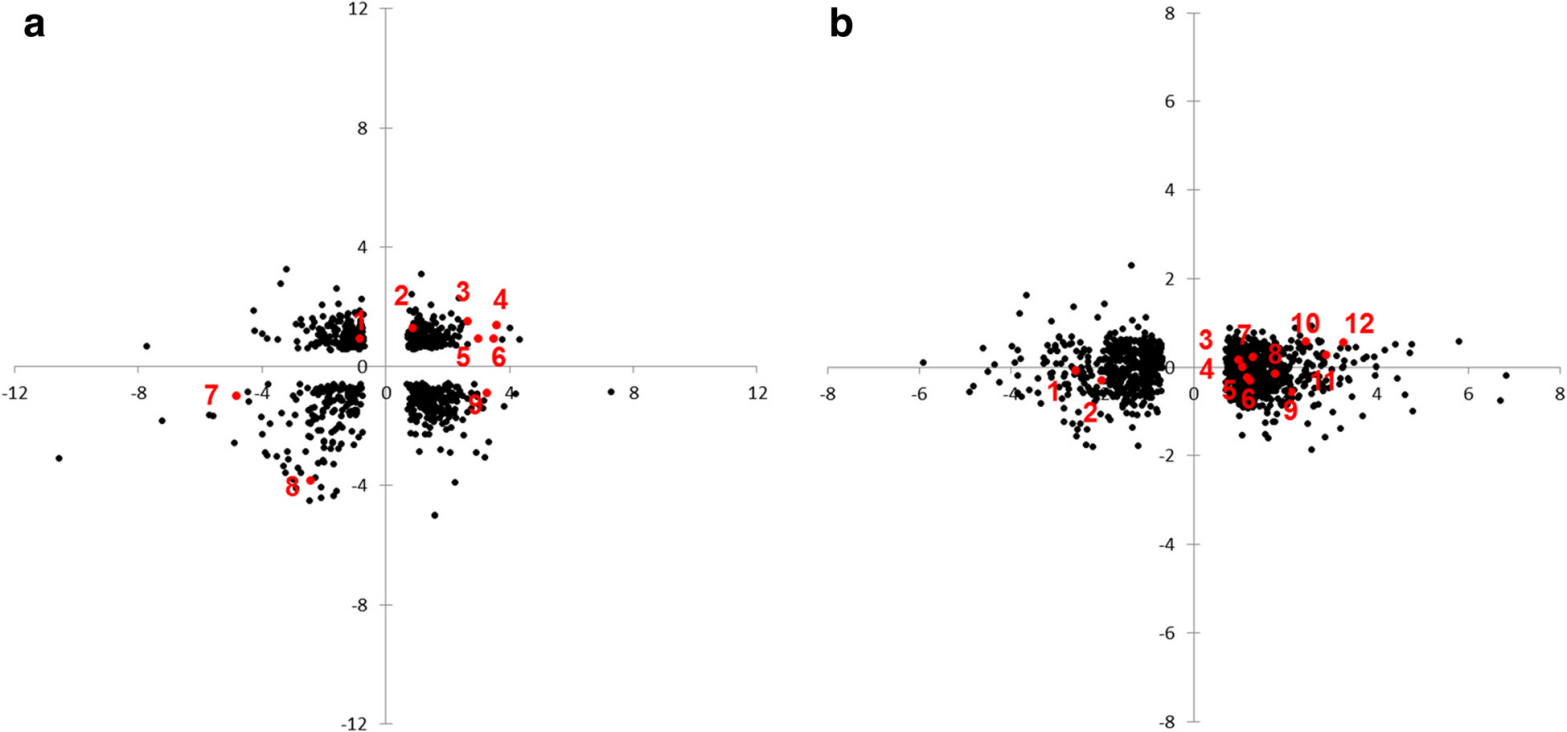
Comparison of *Phlebia* sp. MG-60 and *P. chrysosporium* orthologs. The X-axis indicates log fold change of *Phlebia* sp. MG-60, and the Y-axis indicates log fold change of *P. chrysosporium*. **a**: Orthologous genes with significantly different expression (FDR < 0.05) in *Phlebia* sp. MG-60 and *P. chrysosporium*. Orthologous genes related to glycolysis/gluconeogenesis based on the KEGG pathway database are shown in red, including 1: glucose-6-phosphate 1-epimerase [EC: 5.1.3.15]; 2: phosphoglucomutase [EC: 5.4.2.2]; 3: pyruvate decarboxylase [EC: 4.1.1.1]; 4: phosphoglycerate kinase [EC: 2.7.2.3]; 5: glyceraldehyde 3-phosphate dehydrogenase [EC: 1.2.1.12]; 6: 2,3-bisphosphoglycerate-independent phosphoglycerate mutase [EC: 5.4.2.12]; 7: aldose 1-epimerase [EC: 5.1.3.3]; 8: phosphoenolpyruvate carboxykinase (ATP) [EC: 4.1.1.49]; 9: pyruvate kinase [EC: 2.7.1.40]. **b**: Significantly differentially expressed in *Phlebia* sp. MG-60 (FDR < 0.05) but no significant difference for *P. chrysosporium* (FDR > 0.05) orthologous genes. Orthologous genes related to glycolysis/gluconeogenesis based on the KEGG pathway database are shown in red, including 1: aldehyde dehydrogenase [EC: 1.2.1.3]; 2: fructose-1,6-bisphosphatase I [EC: 3.1.3.11]; 3: pyruvate dehydrogenase E1 component [EC: 1.2.4.1]; 4: glucose-6-phosphate isomerase [EC: 5.3.1.9]; 5: pyruvate dehydrogenase E1 component [EC: 1.2.4.1]; 6: pyruvate dehydrogenase E2 component [EC: 2.3.1.12]; 7: dihydrolipoamide dehydrogenase [EC: 1.8.1.4]; 8: hexokinase [EC: 2.7.1.1]; 9: alcohol dehydrogenase, propanol-preferring [EC: 1.1.1.1]; 10: hexokinase [EC: 2.7.1.1]; 11: fructose-bisphosphate aldolase [EC: 4.1.2.13]; 12: 6-phosphofructokinase [EC: 2.7.1.11]

According to the results of orthologous analysis, 5,391 orthologous gene pairs of *Phlebia* sp. MG-60 and *P. chrysosporium* were acquired. We obtained 729 (FDR < 0.05) orthologous gene pairs of the two fungi, 1,195 significantly differentially expressed in *Phlebia* sp. MG-60 (FDR < 0.05) and none with any significant difference in the *P. chrysosporium* (FDR > 0.05) orthologous gene pairs (Additional file 9: Table S7). Next, we compared the orthologous genes related to glycolysis/gluconeogenesis based on the KEGG pathway database for *Phlebia* sp. MG-60 and *P. chrysosporium*. As shown in Fig. 2a, five orthologous genes annotated as glyceraldehyde 3-phosphate dehydrogenase, phosphoglycerate kinase, pyruvate decarboxylase (PDC), phosphoglucomutase, and 2,3-bisphosphoglycerate-independent phosphoglycerate mutase by BLAST search were consistently upregulated, and two orthologous genes annotated aldose 1-epimerase and phosphoenolpyruvate carboxykinase (ATP) were downregulated in both *Phlebia* sp. MG-60 and *P. chrysosporium*. Two orthologous genes showed different expression levels in *Phlebia* sp. MG-60 and *P. chrysosporium*, and only a pyruvate kinase gene was particularly upregulated in *Phlebia* sp. MG-60. Twelve orthologous gene pairs, including genes encoding aldehyde dehydrogenase, fructose-1,6-bisphosphatase, pyruvate dehydrogenase E1 component (two genes), glucose-6-phosphate isomerase, pyruvate dehydrogenase E2 component, dihydrolipoamide dehydrogenase, hexokinase (two genes), alcohol dehydrogenase (ADH), fructose-bisphosphate aldolase, and 6-phosphofructokinase, were significantly differentially expressed in *Phlebia* sp. MG-60 (FDR < 0.05), with no significant difference in *P. chrysosporium* (FDR > 0.05) (Fig. 2b).

### Semi-quantitative RT-PCR and qRT-PCR of *Phlebia* sp. MG-60

**Fig. 3:**
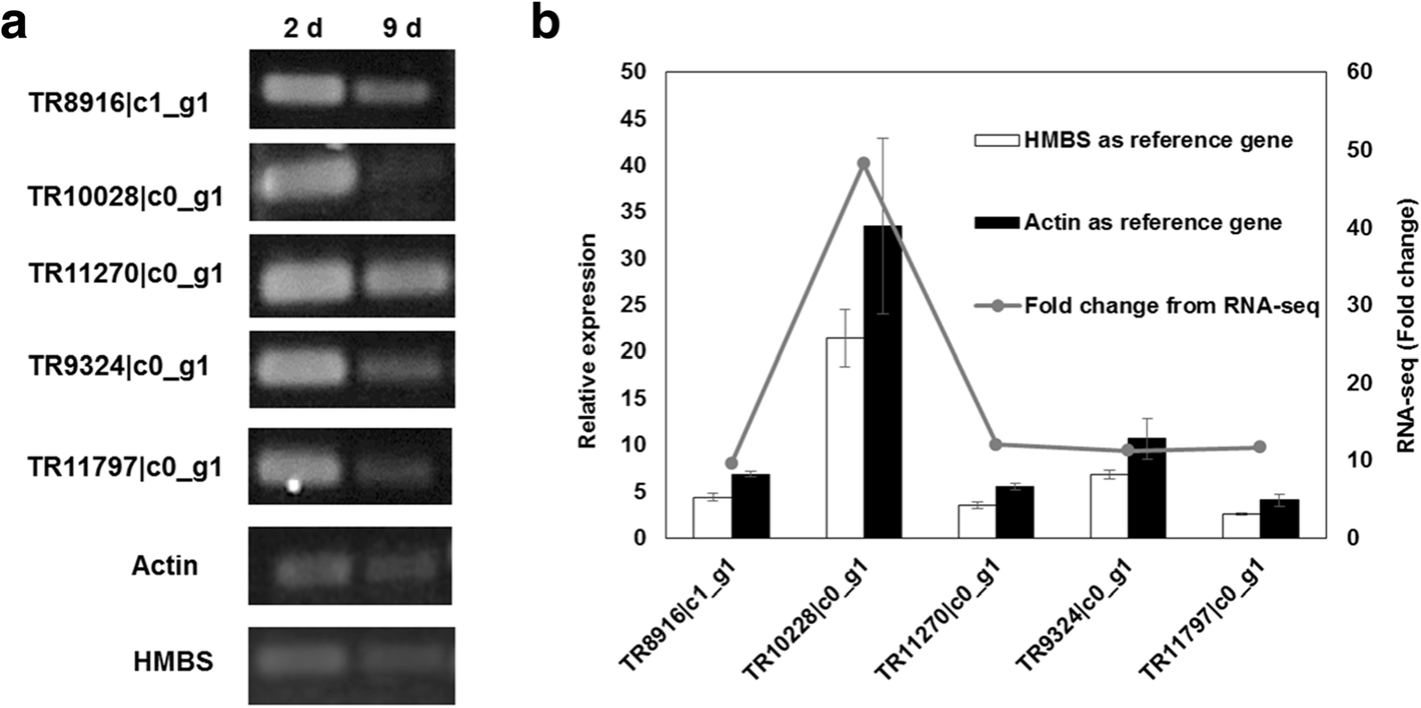
Confirmation of MiSeq results by qRT-PCR (**a**) and RT-PCR (**b**). Five up-regulated genes of *Phlebia* sp. MG-60 at 2 days of incubation were selected for confirmation. Actin and hydroxymethylbilane synthase genes were used as reference genes for qRT-PCR. Values are the means ± SD of triplicate samples

To validate the reliability of the expression profiles obtained by RNA-seq, expression levels of five highly expressed genes related to ethanol fermentation in *Phlebia* sp. MG-60, including one sugar transporter (TR10028|c0_g1), three genes mapped to the glycolysis/gluconeogenesis pathway (TR8916|c1_g1, TR11270|c0_g1, TR9324|c0_g1), and one ADH (TR11797|c0_g1), were compared by semi-quantitative RT-PCR and qRT-PCR. As shown in Fig. 3a, semi-quantitative RT-PCR indicated that all of these genes showed higher expression at 2 days of incubation. Using qRT-PCR, we examined the expression of these genes at 2 and 9 days of incubation and normalized expression to the actin and HMBS genes (Fig. 3b). These results all indicated that expression levels measured by semi-quantitative RT-PCR and qRT-PCR correlated with the RNA-seq analysis.

## Discussion

In this study, we analyzed differential gene expression of the ethanol producing white-rot fungus *Phlebia* sp. MG-60 under fermenting and non-fermenting conditions using next-generation sequencing techniques, and identified the genes involved in its high ethanol production.

### Gene expression of ligninolytic enzymes in *Phlebia* sp. MG-60

Biological delignification is currently attracting much attention as an alternative technology to traditional physicochemical methods for the saccharification of lignocellulosic biomass [3]. White-rot fungi have a unique ability to degrade lignin via extracellular ligninolytic enzymes such as lignin peroxidase, manganese peroxidase (MnP), and laccase [37]. MnP oxidizes Mn^2+^ to Mn^3+^, and Mn^3+^ acts on monomeric phenol, phenolic lignin dimers, and synthetic lignin [38, 39]. Laccases are a group of multi-copper oxidases, which have the ability to oxidize both phenolic and non-phenolic lignin units [40, 41]. Following BLASTx searches of *Phlebia* sp. MG-60 against amino acid sequences in the Swiss-Prot database, thirteen MnP genes and eleven laccase genes were detected (Additional file 4: Table S3). Recently, MnP gene transformants of *Phlebia* sp. MG-60 showed higher MnP activity, and overexpression of the MnP gene improved delignification ability of *Phlebia* sp. MG-60 [42]. The present study was done using glucose as carbon source and therefore not adapted to discuss about ligninolytic enzymes involved in lignocellulose conversion. However, *Phlebia* sp. MG-60 is a candidate for an integrated fungal fermentation process due to its efficient delignification.

### Expression of glucose transporter gene in *Phlebia* sp. MG-60

GO functional enrichment analysis of *Phlebia* sp. MG-60 indicated that two groups, “integral component of membrane” (GO: 0016021) and “membrane” (GO: 0016020), were significantly enriched in the cellular components (Table 2). The first step of sugar metabolism is its transport across the cell membrane [43]. Efficient sugar uptake through the expression of hexose transporter genes can improve fermentation of lignocellulosic biomass to ethanol [44]. Yeast hexose transporters have been most extensively researched, and *Saccharomyces cerevisiae* has at least eight hexose transporters that mediate the uptake of glucose [45, 46]. In the present study, nine glucose transporter genes were expressed in *Phlebia* sp. MG-60. In particular, TR10228|c0_g1, TR10117|c0_g1, TR2714|c0_g1 were characterized as high-affinity glucose transporters, and their expression increased 47.8-, 12.9-, and 4.9-fold under fermenting conditions (Additional file 4: Table S3). Ali et al. reported that the overexpression of a high-affinity glucose transporter gene in the fungus *Fusarium oxysporum* directly affected the glucose and xylose transport capacity and ethanol yield [47]. These results confirm that high-affinity glucose transporter genes expressed at a high level in *Phlebia* sp. MG-60 play an important role in the initiation of ethanol fermentation from glucose.

### Expression of genes involved in glycolysis

**Fig. 4:**
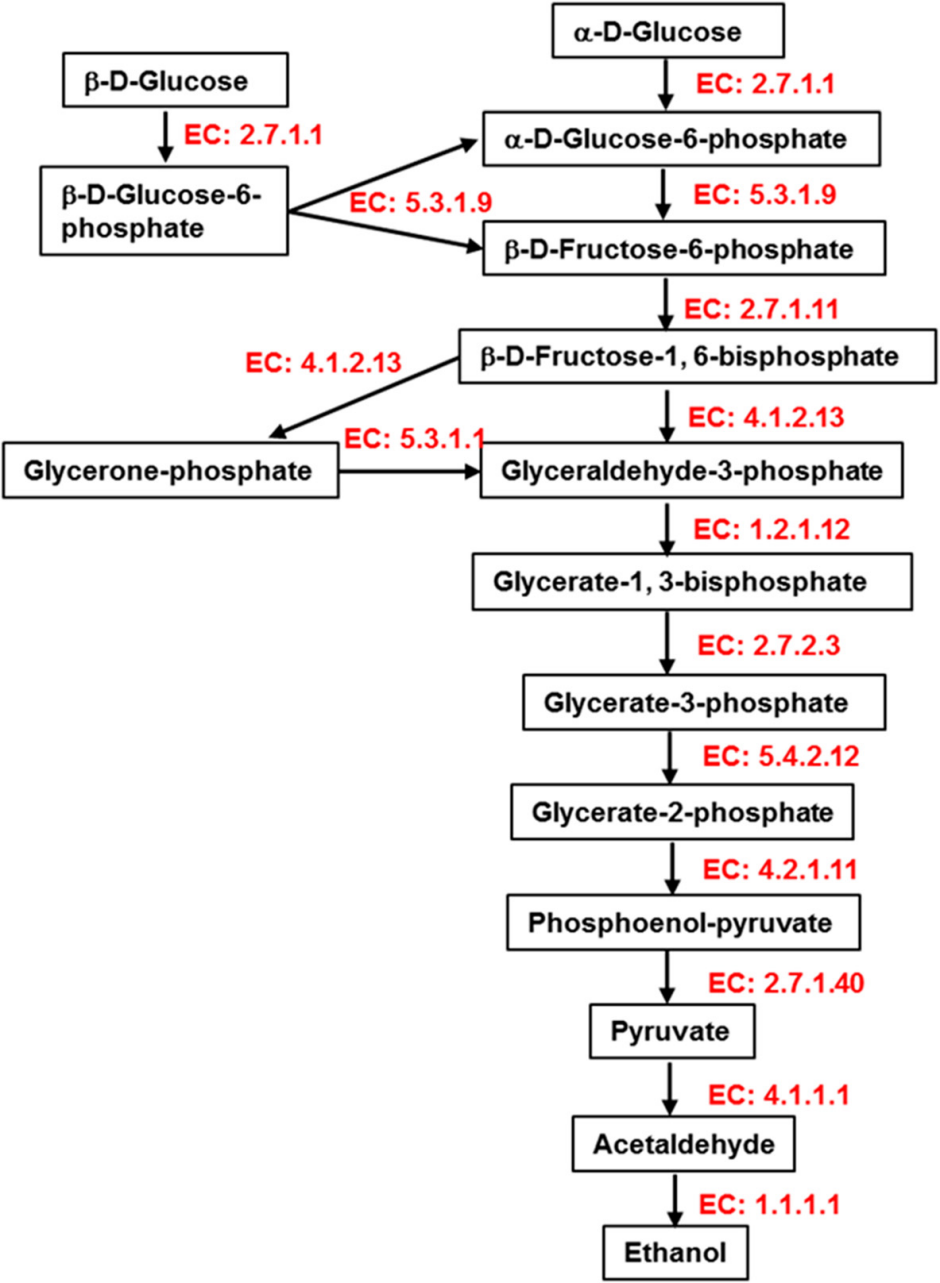
Summary of upregulated DEGs of *Phlebia* sp. MG-60 involved in glycolysis and ethanol fermentation. A list of the identified enzymes is provided in Additional file 7: Table S5

DEGs involved in the glycolysis pathway and ethanol fermentation in *Phlebia* sp. MG-60 are summarized in Fig. 4. For all steps in glycolysis, genes were upregulated in *Phlebia* sp. MG-60 under fermenting conditions, including hexokinase [EC: 2.7.1.1], glucose-6-phosphate isomerase [EC: 5.3.1.9], 6-phosphofructokinase [EC: 2.7.1.11], fructose-bisphosphate aldolase [EC: 4.1.2.13], triosephosphate isomerase [EC: 5.3.1.1], glyceraldehyde 3-phosphate dehydrogenase [EC: 1.2.1.12], phosphoglycerate kinase [EC: 2.7.2.3], 2,3-bisphosphoglycerate-independent phosphoglycerate mutase [EC: 5.4.2.12], enolase [EC: 4.2.1.11], and pyruvate kinase [EC: 2.7.1.40] (Fig. 4, Additional file 7: Table S5). KEGG annotations indicated that all genes involved in the production of ethanol from glucose were expressed in *Phlebia* sp. MG-60 (Additional file 6: Figure S2). In contrast, only nine of these genes were expressed in *P. chrysosporium* (Additional file 6: Figure S2). In current commercial ethanol production, the yeast *S. cerevisiae* is mostly researched, which shows high ethanol yield from glucose, and its prime metabolic pathway of ethanol fermentation is glycolysis [48]. Our results clarified that the ethanol producing white-rot fungus *Phlebia* sp. MG-60 produces ethanol through the same glycolytic pathway, and that it produces ethanol efficiently by upregulating all genes involved in glycolysis.

### Orthologous analysis between *Phlebia* sp. MG-60 and *P. chrysosporium*

To determine whether the expression levels of gene orthologs between *Phlebia* sp. MG-60 and *P. chrysosporium* are correlated, we performed orthologous analysis using FastOrtho. From the results, glyceraldehyde 3-phosphate dehydrogenase [EC: 1.2.1.12], phosphoglycerate kinase [EC: 2.7.2.3], PDC [EC: 4.1.1.1], phosphoglucomutase [EC: 5.4.2.2], and 2,3-bisphosphoglycerate-independent phosphoglycerate mutase [EC: 5.4.2.12], which are important in glycolysis, as shown in Fig. 4, were consistently upregulated in both *Phlebia* sp. MG-60 and *P. chrysosporium* under fermenting condition (Fig. 2a). In particular, almost all log Fold Change (FC) values of upregulated orthologs in *Phlebia* sp. MG-60, except for phosphoglucomutase, were much higher than in *P. chrysosporium*. On the other hand, aldose 1-epimerase [EC: 5.1.3.3] and phosphoenolpyruvate carboxykinase [EC: 4.1.1.49] were downregulated in both species under fermenting condition. Glucose-6-phosphate 1-epimerase [EC: 5.1.3.15] was upregulated in *P. chrysosporium* but downregulated in *Phlebia* sp. MG-60 (Fig. 2a). Although these three enzymes mapped to the glycolysis/gluconeogenesis pathway, they do not play a critical role in glycolysis. Only pyruvate kinase [EC: 2.7.1.40] was upregulated in *Phlebia* sp. MG-60 but downregulated in *P. chrysosporium*. This observation suggested that pyruvate kinase is a rate-limiting enzyme in ethanol fermentation by white-rot fungi. We also compared significantly differentially expressed genes in *Phlebia* sp. MG-60 (FDR < 0.05) and genes that showed no significant difference for orthologous gene pairs in *P. chrysosporium* (FDR > 0.05) (Fig. 2b). Six genes involved in glycolysis and ethanol fermentation were significantly differentially upregulated in *Phlebia* sp. MG-60, including glucose-6-phosphate isomerase [EC: 5.3.1.9], hexokinase [EC: 2.7.1.1] (two genes), fructose-bisphosphate aldolase [EC: 4.1.2.13], 6-phosphofructokinase [EC: 2.7.1.11], and ADH [EC: 1.1.1.1]. Aldehyde dehydrogenase [EC: 1.2.1.3] was differentially downregulated in *Phlebia* sp. MG-60, so it may be related to ethanol production.

### Expression of genes involved in ethanol fermentation via pyruvate

We identified upregulated genes involved in ethanol fermentation via pyruvate in *Phlebia* sp. MG-60 (Fig. 4, Additional file 7: Table S5). Two genes coding for PDC [EC: 4.1.1.1] were identified as DEGs catalyzing the conversion of pyruvate into acetaldehyde and carbon dioxide. Finally, ADH [EC: 1.1.1.1] converts acetaldehyde to ethanol. It is common knowledge that there are two ethanol fermentation pathways from pyruvate. In the two-step ethanol fermentation pathway, pyruvate is non-oxidatively decarboxylated to acetaldehyde by PDC, and then acetaldehyde is converted to ethanol by ADH [49]. In the three-step ethanol fermentation pathway, pyruvate is oxidatively decarboxylated to acetyl-CoA by pyruvate ferredoxin oxidoreductase and pyruvate formate lyase. Acetyl-CoA is then converted to acetaldehyde by a CoA-dependent-acetylating acetaldehyde dehydrogenase. Finally, ADH converts acetaldehyde to ethanol [50, 51]. The three-step pathway is widespread in bacteria, but not in white-rot fungi. In the present study, pyruvate ferredoxin oxidoreductase, pyruvate formate lyase, and CoA-dependent-acetylating acetaldehyde dehydrogenase were not detected in the DEGs of *Phlebia* sp. MG-60 under fermenting conditions. These observations indicated that *Phlebia* sp. MG-60 mainly uses the two-step pathway of ethanol production from pyruvate, and that the PDC and ADH genes play the major roles in ethanol production. Recently, we reported that highly expressing transformants of the PDC gene in the white-rot fungus *P. sordida* YK-624 showed improved ethanol production [52]. As shown in Additional file 7: Table S5 and Additional file 8: Table S6, two genes of PDC in *Phlebia* sp. MG-60 increased PDC expression by 5.0- and 6.3-fold, and three ADH genes increased ADH expression by 11.6-, 4.4-, and 4.1-fold under fermenting conditions. Although six ADH genes were identified in this study, three were downregulated under fermenting conditions. These three upregulated ADH genes may have been the main ones used for ethanol production in the fermentation conditions of this study. In contrast, PDC expression was 2.9- and 3.0-fold increased, and ADH expression was 2.9-fold decreased in *P. chrysosporium* based on RNA-seq data. Additionally, eleven genes for aldehyde dehydrogenase [EC: 1.2.1.3], which is responsible for the subsequent oxidation of acetaldehyde into acetate, were identified in *Phlebia* sp. MG-60. However, all aldehyde dehydrogenase genes of *Phlebia* sp. MG-60 showed low expression in fermentation conditions (Additional file 4: Table S3), which suggested that low expression of aldehyde dehydrogenase in *Phlebia* sp. MG-60 might be a cause of high ethanol productivity.

During ethanol fermentation, the ethanol produced inhibits the growth and viability of the microorganism [53, 54]. Thus, a high level of ethanol tolerance is considered to be important for a high yield of ethanol. It has been reported that alteration in ethanol tolerance could be affected by many factors, such as fatty acid composition and activity of plasma membrane H^+^-ATPase [55–58]. In this study, “fatty acid biosynthetic process” (GO: 0006633) in the biological processes category and “ATP binding” (GO: 0005524) in the molecular functions were the most abundant terms in GO enrichment analysis of *Phlebia* sp. MG-60 under fermenting conditions (Table 2). These observations indicated that a high resistance of *Phlebia* sp. MG-60 to ethanol stress might have an effect on its high ethanol productivity.

## Conclusions

In this study, we provided novel transcriptomic information on the ethanol producing white-rot fungus *Phlebia* sp. MG-60, and investigated the genes involved in its high ethanol yield by comparing them to the model white-rot fungus *P. chrysosporium*. Based on differential gene expression analysis, the genes involved in glucose transport, glycolysis, and ethanol fermentation were upregulated in *Phlebia* sp. MG-60. This suggests that the high ethanol productivity of *Phlebia* sp. MG-60 is due to genes related to glucose uptake, metabolism, including the production of pyruvate, and ethanol synthesis, which are upregulated under fermenting conditions. This study may lead to a better understanding of fermentation by white-rot fungi, and will provide information needed for the genetic engineering of ethanol production.

## Abbreviations

ADH, alcohol dehydrogenase; ATP, phosphoenolpyruvate carboxykinase; DEGs, Differentially expressed genes; FDR, False Discovery Rate; GO, Gene ontology; HMBS: *hydroxymethylbilane synthase*; HPLC, high-performance liquid chromatography; KEGG, Kyoto Encyclopedia of Genes and Genomes; MnP, manganese peroxidase; ORFs, Open reading frames; PDA, potato dextrose agar; PDC, pyruvate decarboxylase; qRT-PCR, quantitative real-time reverse transcription PCR; RNA-seq, RNA-sequencing

## Additional files

Additional file 1: Table S1. Primers used for qRT-PCR and semi-quantitative RT-PCR. (DOCX 29 kb) Additional file 2: Figure S1. Time courses of mycelial growth and pH changes in liquid cultures of *Phlebia* sp. MG-60 (A) and *P. chrysosporium* (B). (PPTX 153 kb) Additional file 3: Table S2. Number of sequencing reads. (DOCX 23 kb) Additional file 4: Table S3. Data for *Phlebia* sp. MG-60 transcripts. (XLSX 3497 kb) Additional file 5: Table S4. Data for *P. chrysosporium* transcripts. (XLSX 2407 kb) Additional file 6: Figure S2. Transcripts of *Phlebia* sp. MG-60 and of *P. chrysosporium* mapped to the glycolysis/gluconeogenesis pathway based on the KEGG pathway database. A list of the identified enzymes is provided in Tables S5 and S6. Genes showing up-regulation and down-regulation are boxed in red and green, respectively. The number of genes is shown in blue. (DOCX 161 kb) Additional file 7: Table S5. Fold changes of transcripts mapped to the glycolysis/gluconeogenesis pathway of *Phlebia* sp. MG-60 based on KEGG. (DOCX 35 kb) Additional file 8: Table S6. Fold changes of transcripts mapped to the glycolysis/gluconeogenesis pathway of *P. chrysosporium* based on KEGG. (DOCX 31 kb) Additional file 9: Table S7. Orthologous analysis of *Phlebia* sp. MG-60 and *P. chrysosporium. (XLSX 6512 kb)*
