# Supplementary material for: Analysis of ethanol fermentation mechanism of ethanol producing white-rot fungus Phlebia sp. MG-60 by RNA-seq
Source: BMC Genomics. 2016 Aug 11;17:616. doi: 10.1186/s12864-016-2977-7 (PMC4982002; doi:10.1186/s12864-016-2977-7)
Supplement: Additional file 1: Table S1. — Primers used for qRT-PCR and semi-quantitative RT-PCR. (DOCX 29 kb) [file 12864_2016_2977_MOESM1_ESM.docx]

Table S1. Primers used for qRT-PCR and semi-quantitative RT-PCR.

| **Gene id** | **Gene description** | **E-value** | **Primer name** | **Nucleotide sequence**  **(5′-sequence-3′)** |
| --- | --- | --- | --- | --- |
| TR8916\|c1_g1 | pyruvate kinase | 0 | TR8916-F | catcatgaggacggcgttga |
|  |  |  | TR8916-R | gctcgagtccatgacctac |
| TR10228\|c0_g1 | high-affinity glucose transporter | 7.00E-138 | TR10228-F | ccagaagttagccttgacctg |
|  |  |  | TR10228-R | gcgttgaacaacaaggccatc |
| TR11270\|c0_g1 | phosphoglycerate kinase | 0 | TR11270-F | gaagctcaagagaggcacct |
|  |  |  | TR11270-R | ttcgaagtcgctcctcgatg |
| TR9324\|c0_g1 | 2,3-bisphosphoglycerate-independent phosphoglycerate mutase | 0 | TR9324-F | ggaggttggtcaccttaaca |
|  |  |  | TR9324-R | gaagtggagacgaccgttag |
| TR11797\|c0_g1 | NAD-dependent alcohol dehydrogenase | 6.00E-28 | TR11797-F | tgcgtaggtgcgagagtatg |
|  |  |  | TR11797-R | tccacgaccttacaccacaa |
| TR11561\|c0_g1 | actin | 0 | actin-F | acgacttgatgtacaccggc |
|  |  |  | actin-R | cggcaatgctgttcagcttg |
| TR7126\|c2_g1 | hydroxymethylbilane synthanse | 3.00E-83 | HMBS-F | gttccactcagcatgccttc |
|  |  |  | HMBS-R | tgacgtgtcgaaggatacag |
