# Supplementary material for: Analysis of ethanol fermentation mechanism of ethanol producing white-rot fungus Phlebia sp. MG-60 by RNA-seq
Source: BMC Genomics. 2016 Aug 11;17:616. doi: 10.1186/s12864-016-2977-7 (PMC4982002; doi:10.1186/s12864-016-2977-7)
Supplement: Additional file 2: Figure S1. — Time courses of mycelial growth and pH changes in liquid cultures of Phlebia sp. MG-60 (A) and P. chrysosporium (B). (PPTX 153 kb) [file 12864_2016_2977_MOESM2_ESM.pptx]

## Slide 1
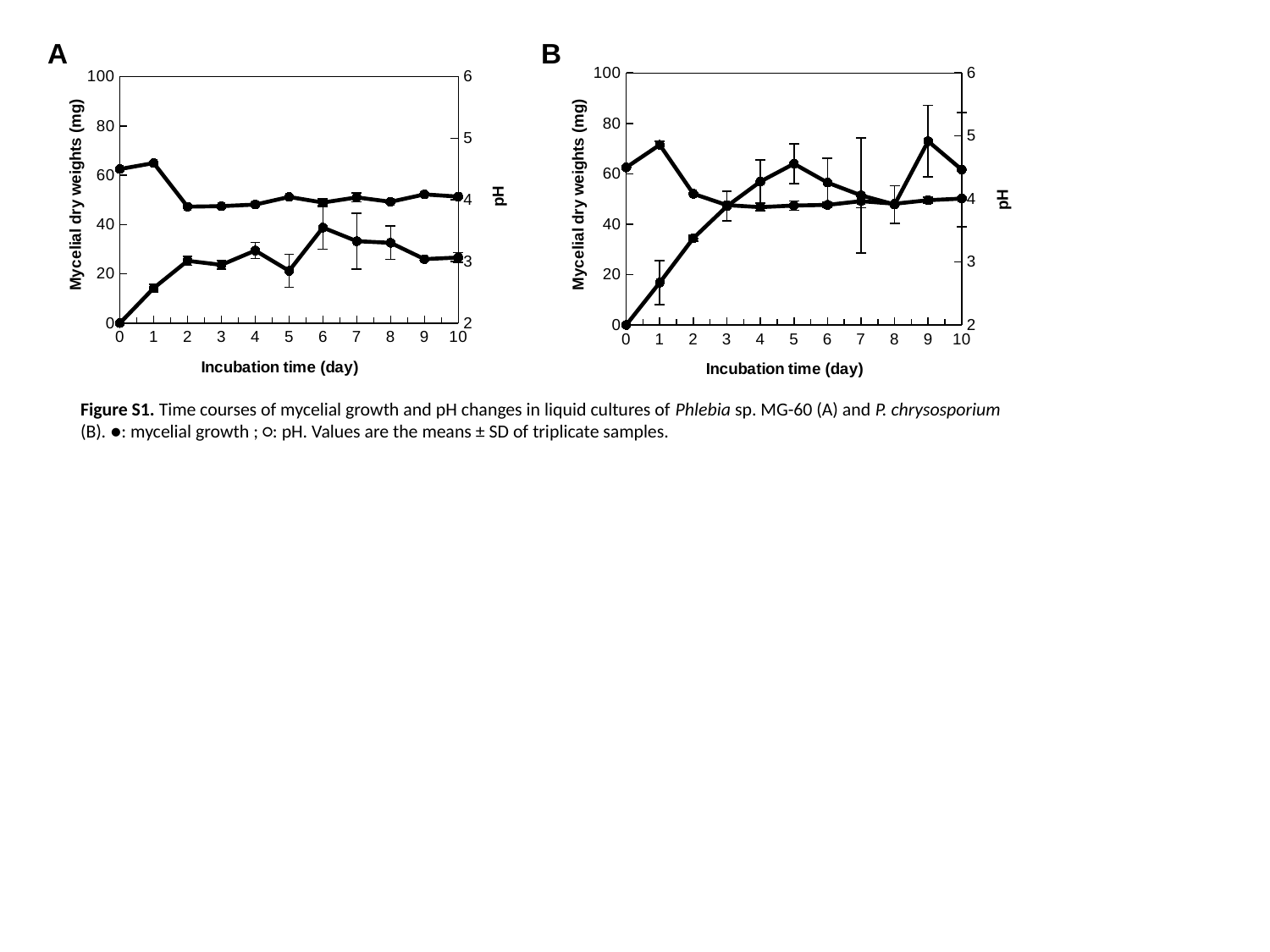

A
B
### Chart
| Category | Fungus growth | pH |
|---|---|---|
| 0 | 0.0 | 4.5 |
| 1 | 16.8 | 4.859333333333334 |
| 2 | 34.4 | 4.082666666666667 |
| 3 | 47.1 | 3.9019999999999997 |
| 4 | 56.9 | 3.8699999999999997 |
| 5 | 63.96666666666667 | 3.895666666666667 |
| 6 | 56.5 | 3.906 |
| 7 | 51.43333333333333 | 3.9653333333333336 |
| 8 | 47.79999999999999 | 3.9243333333333332 |
| 9 | 72.9 | 3.9803333333333337 |
| 10 | 61.63333333333333 | 4.006 |
### Chart
| Category | Fungus growth | pH |
|---|---|---|
| 0 | 0.0 | 4.5 |
| 1 | 14.200000000000001 | 4.597666666666666 |
| 2 | 25.266666666666666 | 3.889666666666667 |
| 3 | 23.633333333333336 | 3.8983333333333334 |
| 4 | 29.433333333333337 | 3.923666666666667 |
| 5 | 21.2 | 4.048 |
| 6 | 38.766666666666666 | 3.956666666666667 |
| 7 | 33.233333333333334 | 4.040333333333334 |
| 8 | 32.56666666666666 | 3.969 |
| 9 | 25.96666666666667 | 4.089333333333333 |
| 10 | 26.599999999999998 | 4.053666666666667 |Figure S1. Time courses of mycelial growth and pH changes in liquid cultures of Phlebia sp. MG-60 (A) and P. chrysosporium (B). ●: mycelial growth ; ○: pH. Values are the means ± SD of triplicate samples.
