# Supplementary material for: Analysis of ethanol fermentation mechanism of ethanol producing white-rot fungus Phlebia sp. MG-60 by RNA-seq
Source: BMC Genomics. 2016 Aug 11;17:616. doi: 10.1186/s12864-016-2977-7 (PMC4982002; doi:10.1186/s12864-016-2977-7)
Supplement: Additional file 3: Table S2. — Number of sequencing reads. (DOCX 23 kb) [file 12864_2016_2977_MOESM3_ESM.docx]

Table S2. Number of sequencing reads.

|  | **Number of raw reads** | **Number of high-quality reads** |
| --- | --- | --- |
| ***Phlebia* sp. MG-60** |  |  |
| **MG2D-read1** | 8,911,385 | 8,712,232 |
| **MG2D-read2** | 8,911,385 | 8,712,232 |
| **MG2D total** | 17,822,770 | 17,424,464 |
| **MG9D-read1** | 12,624,543 | 12,241,172 |
| **MG9D-read2** | 12,624,543 | 12,241,172 |
| **MG9D total** | 25,249,086 | 24,482,344 |
| ***P. chrysosporium*** |  |  |
| **PC3D-read1** | 15,905,252 | 15,411,462 |
| **PC3D-read2** | 15,905,252 | 15,411,462 |
| **PC3D total** | 31,810,504 | 30,822,924 |
| **PC9D-read1** | 13,555,893 | 13,140,075 |
| **PC9D-read2** | 13,555,893 | 13,140,075 |
| **PC3D total** | 27,111,786 | 26,280,150 |
