# Supplementary material for: Analysis of ethanol fermentation mechanism of ethanol producing white-rot fungus Phlebia sp. MG-60 by RNA-seq
Source: BMC Genomics. 2016 Aug 11;17:616. doi: 10.1186/s12864-016-2977-7 (PMC4982002; doi:10.1186/s12864-016-2977-7)
Supplement: Additional file 6: Figure S2. — Transcripts of Phlebia sp. MG-60 and of P. chrysosporium mapped to the glycolysis/gluconeogenesis pathway based on the KEGG pathway database. A list of the identified enzymes is provided in Tables S5 and S6. Genes showing up-regulation and down-regulation are boxed in red and green, respectively. The number of genes is shown in blue. (DOCX 161 kb) [file 12864_2016_2977_MOESM6_ESM.docx]

Figure S2. Transcripts of *Phlebia* sp. MG-60 and of *P. chrysosporium* mapped to the glycolysis/gluconeogenesis pathway based on the KEGG pathway database. A list of the identified enzymes is provided in Tables S5 and S6. Genes showing up-regulation and down-regulation are boxed in red and green, respectively. The number of genes is shown in blue.
