# Supplementary material for: Analysis of ethanol fermentation mechanism of ethanol producing white-rot fungus Phlebia sp. MG-60 by RNA-seq
Source: BMC Genomics. 2016 Aug 11;17:616. doi: 10.1186/s12864-016-2977-7 (PMC4982002; doi:10.1186/s12864-016-2977-7)
Supplement: Additional file 7: Table S5. — Fold changes of transcripts mapped to the glycolysis/gluconeogenesis pathway of Phlebia sp. MG-60 based on KEGG. (DOCX 35 kb) [file 12864_2016_2977_MOESM7_ESM.docx]

Table S5. Fold changes of transcripts mapped to the glycolysis/gluconeogenesis pathway of *Phlebia* sp. MG-60 based on KEGG.

| **KEGG orthology** | **Gene description** | **Gene id** | **LogFold change** | ***P*-value** | **FDR** |
| --- | --- | --- | --- | --- | --- |
| K00121 | alcohol dehydrogenase [EC: 1.1.1.1] | TR1432\|c0_g1 | -1.89 | 8.38E-11 | 3.13E-09 |
| K00128 | aldehyde dehydrogenase [EC: 1.2.1.3] | TR5736\|c0_g1 | -8.36 | 6.15E-16 | 5.13E-14 |
|  |  | TR6724\|c0_g2 | -6.11 | 2.86E-08 | 5.92E-07 |
|  |  | TR6724\|c0_g1 | -4.38 | 9.92E-08 | 1.82E-06 |
|  |  | TR5736\|c1_g1 | -1.34 | 7.77E-05 | 0.000659 |
|  |  | TR8616\|c1_g2 | -1.21 | 7.42E-05 | 0.000636 |
|  |  | TR11189\|c0_g1 | -1.14 | 7.69E-05 | 0.000654 |
|  |  | TR5736\|c0_g2 | -1.14 | 0.011055 | 0.042073 |
| K00134 | glyceraldehyde 3-phosphate dehydrogenase [EC: 1.2.1.12] | TR9150\|c0_g1 | 2.98 | 1.21E-10 | 4.34E-09 |
| K00162 | pyruvate dehydrogenase E1 component [EC: 1.2.4.1] | TR2590\|c1_g1 | 1.16 | 5.49E-05 | 0.000491 |
|  |  | TR2590\|c2_g1 | 1.79 | 9.02E-07 | 1.32E-05 |
| K00382 | dihydrolipoamide dehydrogenase [EC: 1.8.1.4] | TR3338\|c0_g1 | 1.28 | 1.96E-06 | 2.62E-05 |
| K00627 | pyruvate dehydrogenase E2 component (dihydrolipoamide acetyltransferase) [EC: 2.3.1.12] | TR11419\|c0_g1 | 1.23 | 2.50E-05 | 0.000245 |
| K00844 | hexokinase [EC: 2.7.1.1] | TR12467\|c0_g1 | 1.78 | 1.52E-07 | 2.68E-06 |
|  |  | TR9065\|c0_g1 | 2.45 | 1.30E-10 | 4.63E-09 |
| K00850 | 6-phosphofructokinase [EC: 2.7.1.11] | TR10145\|c0_g1 | 3.27 | 4.97E-13 | 2.66E-11 |
| K00873 | pyruvate kinase [EC: 2.7.1.40] | TR8916\|c1_g1 | 3.26 | 1.24E-12 | 6.18E-11 |
| K00927 | phosphoglycerate kinase [EC: 2.7.2.3] | TR11270\|c0_g1 | 3.58 | 1.25E-14 | 8.53E-13 |
| K01568 | pyruvate decarboxylase [EC: 4.1.1.1] | TR12478\|c1_g1 | 2.34 | 2.91E-10 | 9.66E-09 |
|  |  | TR12478\|c0_g1 | 2.65 | 2.07E-12 | 9.99E-11 |
| K01610 | phosphoenolpyruvate carboxykinase [EC: 4.1.1.49] | TR7768\|c2_g1 | -4.84 | 1.33E-17 | 1.41E-15 |
| K01624 | fructose-bisphosphate aldolase [EC: 4.1.2.13] | TR12692\|c0_g1 | 2.88 | 2.67E-11 | 1.09E-09 |
| K01689 | enolase [EC: 4.2.1.11] | TR11340\|c0_g1 | 3.04 | 2.75E-10 | 9.14E-09 |
| K01785 | aldose 1-epimerase [EC: 5.1.3.3] | TR3789\|c0_g1 | -2.42 | 6.22E-09 | 1.51E-07 |
| K01792 | glucose-6-phosphate 1-epimerase [EC: 5.1.3.15] | TR10493\|c0_g1 | -0.84 | 0.002702 | 0.013341 |
| K01803 | triosephosphate isomerase [EC: 5.3.1.1] | TR11619\|c0_g1 | 2.47 | 8.68E-09 | 2.04E-07 |
| K01810 | glucose-6-phosphate isomerase [EC: 5.3.1.9] | TR12733\|c1_g1 | 1.06 | 0.001901 | 0.009916 |
| K01835 | phosphoglucomutase [EC: 5.4.2.2] | TR9090\|c0_g1 | 0.88 | 0.004245 | 0.019255 |
|  |  | TR9090\|c1_g1 | 1.14 | 0.000118 | 0.000945 |
| K03841 | fructose-1,6-bisphosphatase [EC: 3.1.3.11] | TR6381\|c0_g2 | -2.02 | 5.94E-10 | 1.83E-08 |
| K13953 | alcohol dehydrogenase [EC: 1.1.1.1] | TR7827\|c1_g1 | -2.35 | 3.99E-17 | 3.91E-15 |
|  |  | TR7827\|c3_g1 | -1.69 | 3.75E-08 | 7.62E-07 |
|  |  | TR7827\|c0_g2 | 2.05 | 4.25E-06 | 5.18E-05 |
|  |  | TR7827\|c0_g1 | 2.14 | 2.51E-10 | 8.39E-09 |
|  |  | TR11797\|c0_g1 | 3.53 | 1.09E-19 | 1.65E-17 |
| K14085 | aldehyde dehydrogenase [EC: 1.2.1.3] | TR4777\|c1_g1 | -9.38 | 1.53E-06 | 2.11E-05 |
|  |  | TR4777\|c1_g2 | -4.59 | 1.53E-19 | 2.27E-17 |
|  |  | TR4777\|c1_g3 | -2.61 | 1.09E-10 | 3.97E-09 |
|  |  | TR4777\|c0_g1 | -2.58 | 7.45E-12 | 3.31E-10 |
| K15633 | 2,3-bisphosphoglycerate-independent phosphoglycerate mutase [EC: 5.4.2.12] | TR9324\|c0_g1 | 3.48 | 3.96E-12 | 1.82E-10 |

FDR: False discovery rate; Log Fold change values of transcripts upregulated in fermenting condition is represented by positive numbers and downregulated is represented by negative numbers.
