# Supplementary material for: Analysis of ethanol fermentation mechanism of ethanol producing white-rot fungus Phlebia sp. MG-60 by RNA-seq
Source: BMC Genomics. 2016 Aug 11;17:616. doi: 10.1186/s12864-016-2977-7 (PMC4982002; doi:10.1186/s12864-016-2977-7)
Supplement: Additional file 8: Table S6. — Fold changes of transcripts mapped to the glycolysis/gluconeogenesis pathway of P. chrysosporium based on KEGG. (DOCX 31 kb) [file 12864_2016_2977_MOESM8_ESM.docx]

| **KEGG orthology** | **Gene description** | **Gene id** | **Log_2_ Fold change** | ***P*-value** | **FDR** |
| --- | --- | --- | --- | --- | --- |
| K00128 | aldehyde dehydrogenase [EC: 1.2.1.3] | TR6313\|c0_g1 | -4.40 | 7.87E-25 | 6.71E-22 |
|  |  | TR7190\|c1_g1 | 1.01 | 0.009597 | 0.037464 |
|  |  | TR9885\|c2_g1 | 1.78 | 1.35E-12 | 1.05E-10 |
| K00134 | glyceraldehyde 3-phosphate dehydrogenase [EC: 1.2.1.12] | TR1594\|c0_g2 | 0.93 | 0.000664 | 0.004279 |
| K00850 | 6-phosphofructokinase [EC: 2.7.1.11] | TR6983\|c0_g1 | 0.87 | 0.000926 | 0.005592 |
| K00873 | pyruvate kinase [EC: 2.7.1.40] | TR3159\|c0_g1 | -0.88 | 0.00013 | 0.001091 |
| K01568 | pyruvate decarboxylase [EC: 4.1.1.1] | TR7111\|c0_g1 | 1.53 | 7.72E-07 | 1.36E-05 |
|  |  | TR7111\|c1_g1 | 1.57 | 1.32E-07 | 2.94E-06 |
| K01610 | phosphoenolpyruvate carboxykinase [EC: 4.1.1.49] | TR3111\|c0_g1 | -0.97 | 0.001467 | 0.008177 |
| K01689 | enolase [EC: 4.2.1.11] | TR7543\|c0_g1 | 1.46 | 2.65E-05 | 0.000287 |
| K01785 | aldose 1-epimerase [EC: 5.1.3.3] | TR9360\|c1_g1 | -3.85 | 1.82E-17 | 4.22E-15 |
|  |  | TR5436\|c1_g1 | 0.87 | 0.000755 | 0.004738 |
| K01792 | glucose-6-phosphate 1-epimerase [EC: 5.1.3.15] | TR5005\|c0_g1 | 0.93 | 0.000129 | 0.001078 |
| K01803 | triosephosphate isomerase (TIM) [EC: 5.3.1.1] | TR9806\|c6_g1 | 0.74 | 0.003604 | 0.017027 |
| K01835 | phosphoglucomutase [EC: 5.4.2.2] | TR7978\|c0_g1 | 1.29 | 6.38E-07 | 1.15E-05 |
| K01895 | acetyl-CoA synthetase [EC: 6.2.1.1] | TR6498\|c0_g1 | -2.08 | 3.60E-14 | 3.94E-12 |
| K13953 | alcohol dehydrogenase [EC: 1.1.1.1] | TR7153\|c0_g1 | -1.58 | 3.95E-07 | 7.56E-06 |
| K15633 | 2,3-bisphosphoglycerate-independent phosphoglycerate mutase [EC: 5.4.2.12] | TR7826\|c0_g1 | 0.93 | 0.000646 | 0.004181 |

Table S6. Fold changes of transcripts mapped to the glycolysis/gluconeogenesis pathway of *P. chrysosporium* based on KEGG.

FDR: False discovery rate; Log Fold change values of transcripts upregulated in fermenting condition is represented by positive numbers and downregulated is represented by negative numbers.
